# Supplementary material for: Identification of photoperiod-induced specific miRNAs in the adrenal glands of Sunite sheep (Ovis aries)
Source: Front Vet Sci. 2022 Jul 22;9:888207. doi: 10.3389/fvets.2022.888207 (PMC9354845; doi:10.3389/fvets.2022.888207)
Supplement: Supplementary file 1 [file Data_Sheet_1.ZIP › Supplementary file/miRDeep2 file.zip!/miRDeep2 file/Know_Structure/LP42A3/oar-mir-154b.pdf]

miRBase precursor : oar-mir-154b
Total read count : 3096
oar-miR-154b-5p read count : 2891
oar-miR-154b-3p read count : 200
remaining reads : 5

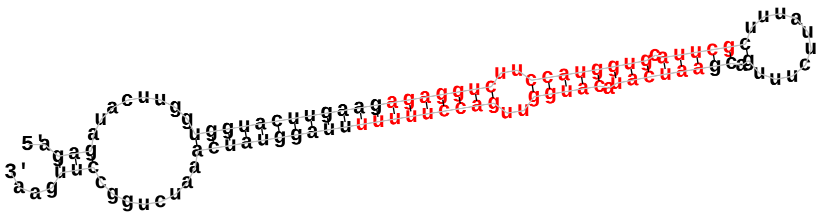

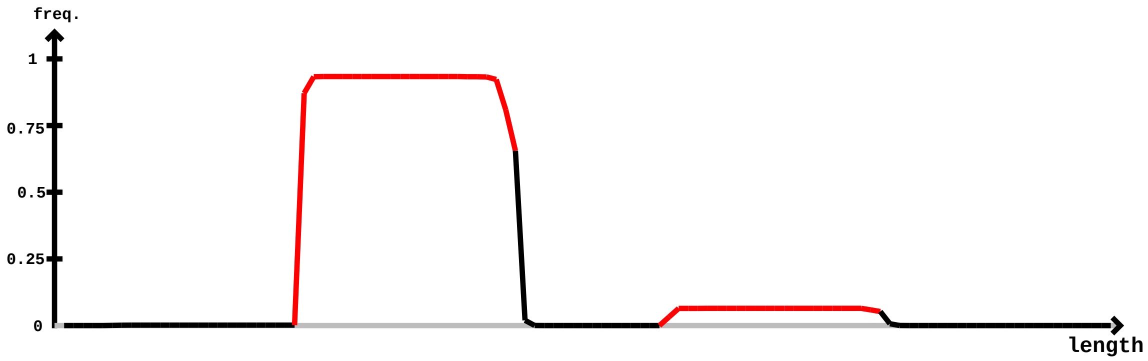


```
                                                                oar-miR-154b-3p
                        oar-miR-154b-5p
5'- agagauacuuggugguacuugaagagaggucuuccauggugcauucgcuuuauucuuugacgaaucauacaugguugaccuuuuuuuagguaucaaaucuggccuugaa  -3'   exp
    .(((........(((((((((((((((((((..((((((((.((((((..........).)))))))).)))))..)))))))))))))))))))........)))...   reads   mm   sample
    ....auacuuggugguacuugaag.....................................................................................   2       0    seq
    .....uacuuggugguacuugaag.....................................................................................   2       0    seq
    ......acuuggugguacuugaaga....................................................................................   1       0    seq
    ........................agaggucuuccauggug....................................................................   1       0    seq
    ........................agaggucuuccaugguA....................................................................   1       1    seq
    ........................agaggucuuccauggugca..................................................................   3       0    seq
    ........................agaggucuuccauggugcau.................................................................   29      0    seq
    ........................agaggucuuccauggugcauu................................................................   346     0    seq
    ........................agaggucuuccauggugcauuc...............................................................   460     0    seq
    ........................agaggucuuccauggugcauucg..............................................................   1805    0    seq
    ........................agaggucuuccauggugcauucgc.............................................................   52      0    seq
    .........................gaggucuuccauggugcauu................................................................   9       0    seq
    .........................gaggucuuccauggugcauuc...............................................................   13      0    seq
    .........................gaggucuuccauggugcauucg..............................................................   163     0    seq
    .........................gaggucuuccauggugcauucgc.............................................................   7       0    seq
    ..........................aggucuuccauggugcauucgc.............................................................   1       0    seq
    ..........................aggucuuccauggugcauucgcu............................................................   1       0    seq
    ..............................................................aaucauacaugguugaAcuuu..........................   2       1    seq
    ..............................................................aaucauacaugguugaccuuu..........................   16      0    seq
    ..............................................................aaucauacaugguugaccuuuu.........................   17      0    seq
    ..............................................................aaucauacaugguugaccuuuuu........................   59      0    seq
    ..............................................................aaucauacaugguugaccuuuuuu.......................   5       0    seq
    ..............................................................aaucauacaugguugaccuuuuuuu......................   1       0    seq
    ...............................................................aucauacaugguugaccuuu..........................   2       0    seq
    ...............................................................aucauacaugguugaccuuuuu........................   82      0    seq
    ...............................................................aucauacaugguugaccuuuuuu.......................   15      0    seq
    ..................................................................auacaugguugaccuuuuu........................   1       0    seq
```
